# Ectomycorrhizal fungal communities associated with *Populus simonii* and *Pinus tabuliformis* in the hilly-gully region of the Loess Plateau, China

**DOI:** 10.1038/srep24336

**Published:** 2016-04-11

**Authors:** Dongfeng Long, Jianjun Liu, Qisheng Han, Xiaobing Wang, Jian Huang

**Affiliations:** 1 College of Forestry, Northwest A&F University, Yangling 712100, Shaanxi, China; 2 College of Landscape Architecture and Arts, Northwest A&F University, Yangling 712100, Shaanxi, China; 3 Ningxia Helan Mountain Forest Ecosystem Orientational Research Station, Yinchuan, 750000, Ningxia, China

## Abstract

The Loess Plateau region of northwestern China has unique geological and dry/semi-dry climate characteristics. However, knowledge about ectomycorrhizal fungal (EMF) communities in the Loess Plateau is limited. In this study, we investigated EMF communities in *Populus simonii* and *Pinus tabuliformis* patches within the forest-steppe zone, in pine forests within the forest zone, and the transitional zone between them. We revealed high species richness (115 operational taxonomic units [OTUs]) of indigenous EMF resources at the Loess Plateau, of which *Tomentella* (35 OTUs), *Inocybe* (16), *Sebacina* (16), and *Geopora* (7) were the most OTU-rich lineages. EMF richness within the forest-steppe zone and the transitional zone was limited, while the natural pine forest maintained diverse EMF communities in the forest zone. The changes of EMF community richness and composition along arid eco-zones were highlighted for the complex factors including precipitation, soil factors, host, DBH, and altitude. Indicator analysis revealed that some EMF showed clear host preference and some taxa, i.e., genera *Geopora* and *Inocybe*, were dominant in drought and alkaline-saline conditions attributed to their environmental preference. This study revealed that EMF communities were quite limited in the forest-steppe zone, while the forest region contained diverse EMF communities in the Loess Plateau.

Ectomycorrhizal symbioses formed between special groups of fungi and plant fine roots, which are essential for plant growth through improving water uptake and nutrient acquisition^1^. In addition, ectomycorrhizal fungi (EMF) can improve their host partner adaptation to diverse stress conditions, such as pathogenesis, heavy-metal toxicity^2^,^3^, drought conditions^4^, and etc. Approximately 20,000–25,000 EMF species are estimated to exist in nature^5^,^6^, and ectomycorrhizal plants are often colonized by diverse EMF species, which comprised the important part of forest ecosystem, woodland system and shrub land. However, EMF community structure is correlated with the plant community status and mediate plant competition, and also influenced by the environmental factors. Under drought conditions, ectomycorrhizae can improve the drought tolerance of plants by enhancing water uptake from soil via huge mycelial networks^7^ and regulating aquaporin function^8^. In the last several decades, regional and local droughts became more frequent due to the global climate change^9^, and the response of EMF communities to drought conditions is consequently drawing much attention.

Although EMF communities in drought/alkaline condition have been concerned, the documents were still limited. Some studies found decreases in EMF diversity with increasing moisture stress^10^,^11^, while others observed contradictory relationships^12^,^13^. In addition, drought may also cause a shift in EMF composition^14^,^15^. Gehring *et al.*^14^ revealed a considerable difference in EMF community composition associated with the pinyon pine (*Pinus edulis*) between a cinder site (drought and infertile) and a sandy-loam site (higher moisture and nutrients)^14^. To improve our understanding of how EMF communities are affected by drought and confounding factors in arid regions, we require more data, especially in Asian regions where few data are available so far.

The Loess Plateau of China (LPC) covers an area of ca. 640,000 km^2^ and is characterized by an arid and semi-arid climate. Long-lasting and large-scale water shortages have resulted in ecosystem degradation and serious soil erosion. Asian dust originating from the degraded LPC seriously affects natural environments and human health in much of East Asia, including China, Korea, and Japan. Natural vegetation zones in the LPC change from forest to desert with the declining precipitation gradient from southeast to northwest^16^. Forest patches and fragmented forests in this region are dominated by ectomycorrhizal host species, such as pine, poplar, oak, and birch. So far, EMF communities have only been investigated in *Quercus liaotungensis* forests within a narrow area^17^,^18^. For a comprehensive understanding of EMF communities in the LPC, the study of EMF communities in other host species from a wider region is needed. Such studies may be important for the reforestation of these degraded sites, given the massive impact of the degrading LPC on nature and human society in East Asia.

In this study, we investigated EMF communities in *Populus simonii* and *Pinus tabuliformis* patches within the forest-steppe zone and pine forests within the transitional and forest zones in the LPC. We attempt to address the following questions: (1) Are there special EMF communities associated with the poplar and Chinese pine in this arid region? (2) How do EMF community composition and structure change from the forest zone to the forest-steppe zone along the precipitation gradient? (3) If EMF community composition does change, which factors determine the EMF community structure in this region?

## Results

### EMF molecular identification

In total, we morphotyped 20,332 ectomycorrhizal tips (9,795 from poplar and 10,537 from pine) from 114 root samples (45 for poplar and 69 for pine). In total, 546 poplar tips and 887 pine tips were subjected to molecular analysis. We selected one well-amplified ITS PCR product from each morphotype in a given root sample for direct sequencing. Finally, we obtained 188 unique sequences (available in the International Sequence Database under accession numbers LC013704–LC013891). These unique sequences were classified into 116 operational taxonomic units (OTUs) (including 1 non-EMF OTU) at the 97% identity level, of which 39 OTUs were associated with poplar and 84 OTUs with pine. In general, 107 OTUs were assigned to known species or genera, while 10 OTUs remained unknown because of low identity with their closest matches. Twenty-four OTUs (7 from poplar, 18 from pine) belonged to Ascomycota and 91 (32 from poplar, 66 from pine) belonged to Basidiomycota. *Tomentella* (35 OTUs), *Inocybe* (16), *Sebacina* (16), and *Geopora* (7) were the most OTU-rich genera, while others had less than five OTUs. As shown in Supplementary Table S1, pine EMF included 19 lineages while only eight lineages were detected from poplar. *Tomentella* was the most OTU-rich genus both in poplar (15) and pine (24). Poplar had more *Inocybe* OTUs (11 vs. 6), but significantly less *Sebacina* OTUs (1 vs. 15) than pine. In total, 38 OTUs were singletons detected in a single root sample, and only eight OTUs were shared between the poplar and Chinese pine.

### EMF community richness

**Figure 1:**
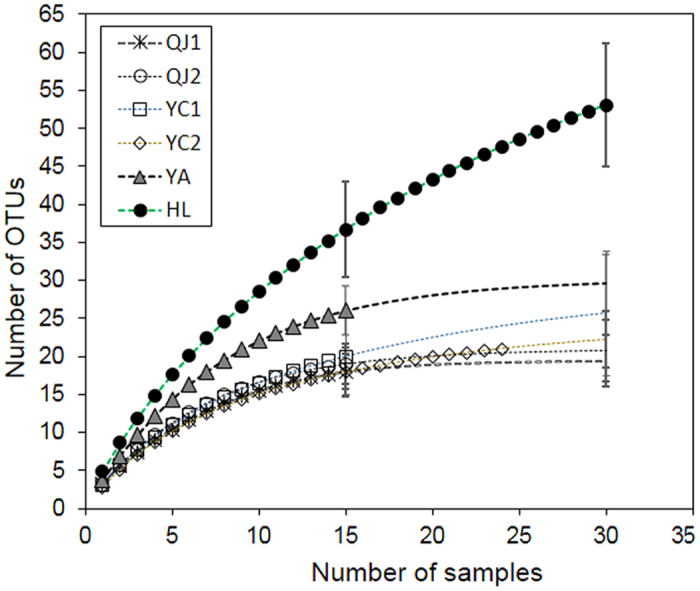
Accumulation curves of rarefied operational taxonomic units (OTUs) and their 95% confidence intervals (lines with terminal bars) at sample sizes of 15 and 30 for six sites. QJ1, QJ2, and YC1 were poplar EMF communities; YC2, YA, and HL were Chinese pine EMF communities.

As shown in Table 1, the three poplar patches showed similar levels of EMF richness per site (18–20) or per root (3.07); the pine root samples from YC2 also had low root richness (2.79 ± 0.88), but the other two pine sites showed higher richness/root sample (YA: 3.60 ± 1.12, HL: 4.90 ± 1.32). The OTU accumulation curves showed that three poplar EMF communities and two pine EMF communities (YC2, YA) nearly reached plateau at the sample size of 30 after extrapolation, and did not differ significantly according to 95% confidence intervals at the sample sizes of 15 or 30. The accumulation curve for the EMF community in HL did not show any sign of reaching asymptote, indicating that far more EMF species would be found with additional sampling (Fig. 1).

### EMF community structure

As listed in Table 1, the three poplar EMF communities had similar Shannon *H*’ values (2.11–2.74), which were also close to the pine EMF community from YC (2.65). In contrast, the HL and YA pine EMF communities had higher values for Shannon *H*’ and the Simpson index 1/D (HL: 3.31 and 13.3; YA: 2.95 and 15.99). In addition, the Bray-Curtis dissimilarity index for each site highly ranged from 0.74–0.93.

**Figure 2:**
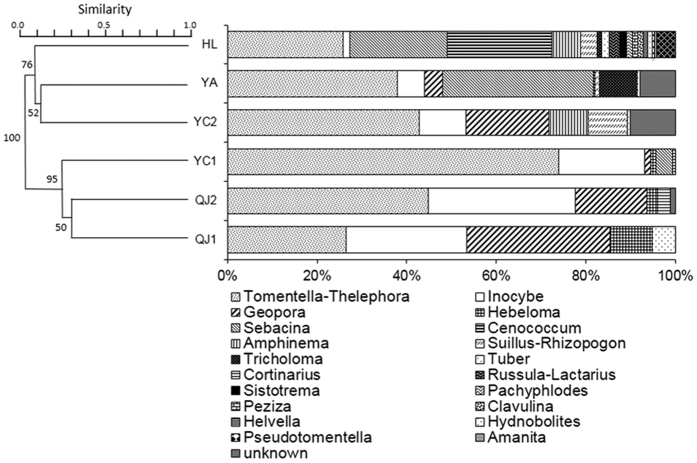
Relative abundances of ectomycorrhizal fungal (EMF) taxa at each site and cluster analysis of the six sites based on EMF compositions using the unweighted pair group method with arithmetic mean (UPGMA) method based on Bray–Curtis distance (bootstrap = 999).

In terms of OTU composition (Supplementary Table S2), the three poplar EMF communities showed high similarity, of which, five OTUs (*Geopora* sp.1, *Inocybe exilis*, *Inocybe* sp. 3, *Tomentella* sp. 11, and *Tomentella* sp. 20) were common among the three sites. In contrast, no OTUs were shared among the three pine EMF communities. The HL pine stand shared more OTUs with YA (10) than with YC2 or the three poplar EMF communities. The Mantel test also revealed that EMF community similarities were significantly correlated with geographic distance (*R* = 0.49, *P* = 0.029). At the higher taxonomic level, as shown in Fig. 2, *Tomentella* and *Inocybe* occurred at all six sites. According to the cluster analysis, the six communities were clustered into two groups, that is, pine and poplar; the three poplar EMF communities clustered together and were dominated by a few lineages. Although the pine EMF communities formed a single cluster, they comprised more diverse EMF lineages, especially in HL.

At the genus level, *Tomentella* was the most abundant taxon at all sites excluding QJ1, where *Geopora* and *Inocybe* were more abundant. As shown in Fig. 2, *Hebeloma* and *Cortinarius* were specific to poplar, while several lineages, such as *Amphinema* and *Cenococcum*, were abundant in pine communities but absent from poplar communities. The poplar EMF communities were overwhelmed by three lineages, *Tomentella*, *Inocybe*, and *Geopora*. In association with poplar, *Tomentella* was the most abundant/frequent lineage, with 15 OTUs, and was found on 41 of 45 trees, followed by *Inocybe* (10 OTUs) on 33 trees and *Geopora* (5 OTUs) on 22 trees. At the species level, *Geopora* sp. 1 was the most frequent OTU, found on 17 trees, followed by *Tomentella* sp. 11 on 12 trees and *Inocybe* sp. 3 on 11 trees. For the pine forests, *Tomentella* (24 OTUs) was again the most abundant/frequent lineage from all three sites. YC2 was similar to the poplar EMF communities, and *Inocybe* and *Geopora* were again abundant with over 10% relative abundance. At YA, however, *Sebacina* was the second most abundant with 33.6% relative abundance. At HL, *Cenococcum* (23.4%) and *Sebacina* (21.6%) were the second and third most abundant taxa, following *Tomentella*.

### Correlation of environmental factors with EMF distribution patterns

**Figure 3:**
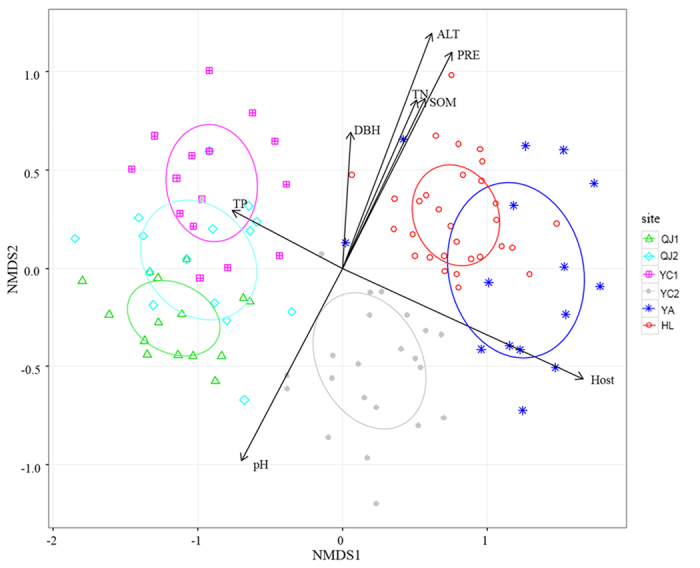
Non-metrical multidimensional scaling (metaNMDS, Stress: 0.061) of OTUs occurring on more than one tree for six sites, and correlation with soil nutrients, climatic foctors and host. Only significant correlations between NMDS structure and factors are shown (*P* < 0.001). PRE, precipitation; ALT, altitude; SOM, soil organic matter content; TN, total nitrogen; TP, total phosphorus.

Canonical correspondence analyses (CCA) were performed to characterize the correlation of environmental factors with the distribution of EMF OTUs. Analyses revealed that five soil factors and altitude explained 19.3% of the variance structuring EMF communities. Environmental fitting tests (permutations = 999) revealed that precipitation, altitude, and four soil parameters (soil organic matter [SOM], total nitrogen [TN], pH, and total phosphorous [TP]) had significant effects on the EMF communities (Table S3). In addition, non-metric multidimensional scaling (NMDS) ordination revealed that pine and poplar EMF communities were clearly separated. Environmental fitting tests (permutations = 999) also revealed that host type, DBH, precipitation, and four soil parameters (SOM, TN, pH, TP) had significant effects on the EMF communities (Fig. 3, Table S4). We also used a indicator species analysis to revealed significant host preference of the EMF-OTUs (Table 2). For example, *Geopora* sp.1, *Tomentella* sp.11, *Inocybe exilis*, *Inocybe* sp.3 significantly preferred *P. simonii* while *Cenococcum geophilum*2, *Suillus luteus* to *P. tabuliformis* (Table 2). Results of EMF–forest type association strength test by indicator species analysis indicated that *C. geophilum*2, *Tomentella* sp.16 significantly preferred forest zones, *Geopora* sp.1, *Tomentella* sp.11 to forest-steppe zones, *Sebacina* sp.9, *Sebacina* sp.4, *T. ferruginea*, *Tomentella* sp.10, *Sebacina* sp.1, *Sebacina* sp.6, *Tricholoma terreum* and *Geopora* sp.3 to the transitional regions (Table S5).

## Discussion

The LPC is known worldwide for its unique geographical conditions and severe soil erosion regions. Ecological restoration is necessary to mitigate water shortages and soil salinization^16^. In the present study, a total of 115 EMF species were identified from *Po. simonii* and *Pi. tabuliformis*, which have never been used for EMF research in this region, greatly improving the local EMF inventory. However, EMF communities in the forest-steppe zone were clearly less diverse than those in the forest zone and the transitional zone in this study, as well as those documented in *Q. liaotungensis* mature forests in the forest region, that is, 70 species from 30 samples^18^ and 135 RFLP types from 81 samples by ITS-RFLP analysis^17^. Poplar and pine patches in the forest-steppe zone exhibited early successional stages, while the pine site at HL and *Q. liaotungensis* reported by Zhang *et al.*^17^,^18^ were in late successional stages. Thus, the LPC may still harbor rich EMF species under drought and alkaline-saline stress when the forests mature.

Vegetation succession is a very complex process, and shows several visible changes, such as the community structure and physiognomy of vegetation. This phenomenon has also been extended to the below-ground EMF communities in temperate forests undergoing primary and secondary succession^19^,^20^ and in subtropical forests undergoing secondary succession^21^. In this study, the below-ground EMF community was also sharply changed among forest successional stage in the LPC region. We found the EMF lineages *Geopora* and *Inocybe* were abundant in poplar forest at the early succession stage, but *Cenococcum* and *Sebacina* were abundant in late-successional pine forest. Similar results were also reported in many other temperate forest^20^,^22^,^23^.

Complex environmental factors changed from the forests zone to the forest-steppe zone with the variation of succession stage, for example, SOM, N, precipitation, and mean temperature increased toward the forest zone, while TP content decreased. In this study, NMDS ordination illustrated that soil nutrients (pH, TN, TP and SOM), DBH and climatic factors (altitude and precipitation) co-influenced the EMF species distribution significantly. In addition, cluster analysis and NMDS ordination showed a clear separation of the pine EMF and poplar EMF communities, which may be due to the host effect. Obviously, a great phylogenetic distance exists between the poplar (angiosperm) and pine (gymnosperm). Although there were not enough site replicates in each ecological zone for either host species, the host effect on the EMF community structure in the arid region should be taken into consideration.

Drought and alkaline-saline conditions were the major soil edaphic characteristics in the LPC, which could potentially affect EMF distribution. Among edaphic variables, Tedersoo *et al.*^24^ pointed out that soil pH was one of the most important predictors of fungal OTU richness^24^. Yamanaka also found that EMF richness was greatest in slightly acidic to neutral soils and that the growth of EMF is depressed at higher pH conditions^25^. In the present study, soil pH ranged from 7.7–8.47, and was especially high in the forest-steppe zone (up to pH 8.67) in the LPC. Although available information about EMF communities in highly alkaline habitats is limited, Ishida *et al.*^26^ identified 11 EMF species in 25 root samples of Mongolian willow (*Salix linearistipularis*) growing in alkaline-saline soil (up to pH 9.2) in northeastern China^26^. Because we did not have enough replicates, it is still uncertain whether or not the difference in EMF richness between the forest zone and the forest-steppe zone observed in this study is related to soil pH.

Among the 115 OTUs identified in this study, lineages *Tomentella* (35 OTUs), *Inocybe* (16), *Sebacina* (16), and *Geopora* (7) were the most OTU-rich lineages. Indicator species analysis revealed that different EMF showed significant host or forest-type preference. Lineages *Geopora* and *Inocybe* significantly preferred *Po. simonii* and forest-steppe zone, while lineage *Cenococcum* to *Pi. tabuliformis* and mature forest zone. Similarly, Ding *et al.* reported that most of common EMF species showed significant preference to host plant species in subtropical evergreen broad-leaved forests^27^.

Lineage *Inocybe* often occur as a pioneer fungi in primary succession sites^28^,^29^ and are also frequently found in disturbed ecological conditions, such as mine wastelands^2^,^3^,^30^ and post-fire forestland^31^. In the present study, *Inocybe* was present at all six sites, however, there was a clear decreasing trend in its relative abundance from north (QJ) to south (HL), which is displayed in Fig. 2. This suggests that lineage *Inocybe* prefers drought conditions, and thus may have important ecological roles in the dry LPC.

In addition, the lineage *Geopora* (7 OTUs) was also abundant in the forest-steppe zone (QJ1, QJ2, and YC2) but disappeared in the forest zone (HL). The mycorrhizal *Geopora* distributed widely in North America, Europe and East Asia, and also involved diverse host plants including coniferous and deciduous, as well as orchid. At the same time, mycorrhizal sequences also revealed *Geopora* species did not show distinct geographical populations^32^. As reviewed by Tamm *et al.*^32^ many *Geopora* species preferred alkaline conditions^32^. Ishida *et al.*^26^ found that *Geopora* also dominated the Mongolian willow root systems in a severe alkaline conditions (up to pH 9.2)^26^. More recently, Gehring *et al.*^33^ observed the abundance of genus *Geopora* positively responded to the high parasitism, high competition, and high herbivory trees^33^. Considering some *Geopora* members were also abundant in the forest-steppe region of the LPC with drought & alkaline soil conditions, *Geopora* could be defined as an important mutual partner for host plant resisting to the stress conditions.

In contrast to lineages *Inocybe* and *Geopora*, a lineage *Cenococcum* was abundant in the forest region (HL), but disappeared in the forest-steppe region (Fig. 2). *C. geophilum* is globally distributed and has been described as a drought-tolerant EMF species^34^, as well as an efficient root protective agent against drought^35^. However, the majority of previous studies have found *C. geophilum* presenting in mature forests. For example, *C. geophilum* was a dominant EMF species in a *Q. liaotungensis* forest on Dongling Mountain^36^ and in a Mediterranean *Quercus ilex* forest^37^. *C. geophilum* depends on sclerotia for dispersal and thus has a limited capability for long distance dispersal^38^. This fact could explain that it may take a long time for *C. geophilum* to reach those fragmented forest patches although *C. geophilum* exists in the LPC.

## Conclusion

We identified several indigenous EMF resources associated with *Po. simonii* and *Pi. tabuliformis* in the LPC, and highlighted the changes in pine EMF community richness and composition along arid eco-zones. Environmental factors, including soil factors, host species, DBH, and precipitation, were significantly related to EMF community structure, although we could not isolate their effects because these conditions are significantly correlated with each other and as well as with successional factors in the region. The preference of some EMF lineages, such as *Geopora* and *Inocybe*, for drier and high pH habitats could contribute to the future applications of these species for improving survival and growth of host plants under drought conditions.

## Methods

### Site description

**Figure 4:**
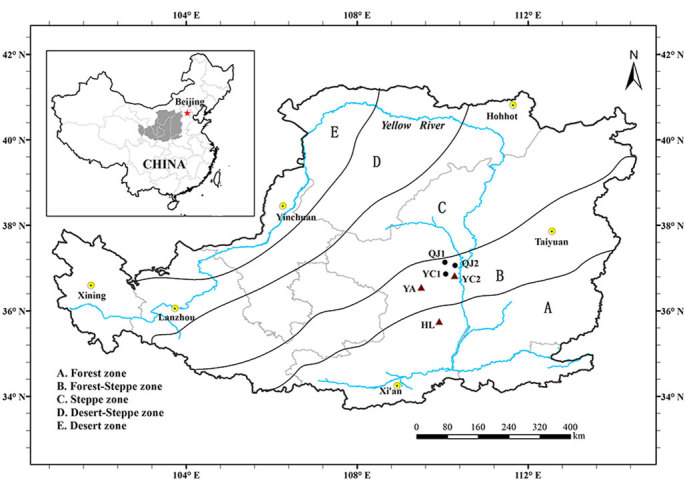
Locations of sampling sites in the Loess Plateau, China. Circles indicate poplar plots; triangles indicate pine plots. Maps generated using Arcgis10.2 (ESRI Inc. 2014).

Sampling sites were selected in three categories of vegetation zones in the LPC, the forest-steppe zone, the forest zone, and the transitional region between the two zones, from the north to south in northern Shaanxi Province, as shown in Fig. 4. These sites were located in a typical hilly-gully region of the LPC. The climate in this region, including the sampling sites, is classified as a temperate semiarid zone. The mean annual temperature ranges from 7.0–10.8 °C, and the mean annual precipitation ranges from 500 to 612 mm (decreasing northward), where most of the rainfall occurs during the rainy season (July to September).

In total, six forest stands were selected for sampling: four stands within the forest-steppe zone, including two sites (QJ1 and QJ2) in Qingjian County and two stands (YC1 and YC2) in Yanchuan County; one stand (YA in Yan’an City) within the transitional zone; and one site (HL in Huanglong County) within the forest zone. In addition, these stands could be classified into two types according to the forest composition: poplar (*Po. simonii* in QJ1, QJ2, and YC1) and Chinese pine (YC2, YA, and HL). The poplar and Chinese pine were the only two ectomycorrhizal tree species used in the afforestation program within the forest-steppe zone, thus we selected these two species in this study.

All selected stands were pure forest, the individuals of *Po. simonii* or *Pi. tabuliformis* represented more than 90% of the total tree individuals, and the other trees made up less than 10%. The associated vegetation at *Po. simonii* sampling sites QJ1, QJ2, and YC1 were mainly various shrubs and herbs, including *Platycladus orientalis*, *Armeniaca sibirica*, *Lonicera* spp., *Spiraea* spp., *Stipa bungeana*, and *Artemisia sacrorum*. In YC2 and YA stands, ground plant species were few; only *Platycladus orientalis* and *Stipa bungeana* occurred. At HL, located in the forest zone, in addition to *P. tabuliformis*, the only other EMF hosts were occasional *Quercus liaotungensis* trees. In the shrub layer, *Rosa xanthina*, *Cotoneaster* spp., *Syringa* spp., *Berberis amurensis*, *Spiraea* spp., and *Sophora davidii* were frequent. On the forest floor there were herb species such as *Bothriochloa ischaemum*, *Pedicularis* spp., *A. sacrorum*, *Artemisia giraldii*, and *S. bungeana*. The detailed information of the six sites, including latitude/longitude, altitude, site history, annual rainfall, and temperature range, are given in Table 3.

### Sampling strategy

We selected 15 poplar trees for collecting fine root systems from sites QJ1, QJ2, and YC1, respectively, and 24, 15, and 30 pine trees from sites YC2, YA, and HL, respectively. At each sampling site, the distance between the selected trees was maintained at >10 m, in an attempt to secure the independence of the samples. From each selected tree, a main root (~15–30 cm in length) with fine root tips was gently collected from a soil depth of 0–30 cm. All root systems were traced from the trunk to confirm the identity of the roots. The rhizosphere soil (~100 mL) was collected for chemical analyses. Meanwhile, the diameter at breast height (DBH) of each sampled tree was measured. Geographical coordinates and altitude were recorded using a Garmin eTrex Venture HC GPS (Garmin International Ltd, Olathe, Kansas, US).

### Ectomycorrhizal morphotyping and molecular identification

After returning to the laboratory, the collected root samples were immediately washed to remove soil particles, and morphotyped under a stereomicroscope (EZ4HD, Leica Microsystems, Germany) as described by Huang *et al.*^3^. Living ectomycorrhizal root tips were counted to determine colonization and then sorted into different morphotypes according to Agerer^39^. Approximately 5–10 replicates of the healthy ectomycorrhizal tips (or all, if fewer than five tips were available) for each morphotype from each root system were randomly selected, individually placed into 2.0-mL tubes and lyophilized for DNA extraction using a freeze dryer (FD5-2.5, Sim International Group Co. Ltd, USA). The total genomic DNA of the dried ectomycorrhizal tips was extracted via a modified cetyltrimethylammonium bromide (CTAB) method according to Huang *et al.*^2^ using a bead beater (MM400, Restch, Germany). Amplification by polymerase chain reaction (PCR) was carried out in 30-μL reactions containing 1.5 μL of template DNA, 15 μL of 2 × Taq MasterMix (Kangwei, Beijing, China), 12.3 μL deionized water, and 0.6 μL fungal-specific primers ITS1-F and ITS4^40^,^41^. PCR cycling parameters were as follows: 94 °C for 5 min; 35 cycles of 94 °C for 30 s, 53 °C for 30 s, and 72 °C for 1 min; and a final 5 min 72 °C extension. The obtained PCR products were verified by electrophoresis on 1.5% agarose gels with the Bio-Rad Gel Doc^TM^ XR + system (Bio-Rad, CA, USA). Then one representative sample with a unique electrophoretic band within a root sample was selected, the electrophoretic band was cut and purified for sequencing using a QIAquick PCR Purification Kit (Qiagen, Inc., Valencia, CA). The direct sequencing of the PCR products using primers ITS1-F/ ITS1/ ITS4 was performed on an ABI Prism 3730xl genetic analyzer (Applied Biosystems, Foster City, CA). Because we found that replicates from the same morphotype within the same root sample almost all had similar band sizes and sequences (sequence identity ≥99.5%), replicates from the same morphotype were usually treated as the same operational taxonomic unit (OTU).

The obtained sequences were first edited and manually corrected in BioEdit 7.0.8 (http://www.mbio.ncsu.edu/BioEdit/bioedit.html). The redundant sequences (the same sequence with the same/different length) were then removed, retaining the unique sequences for further analysis. The unique sequences were clustered into species-level OTUs at the 97% sequence identity threshold using BLASTclust (http://toolkit.tuebingen.mpg.de/blastclust)^42^. To further confirm distance, we performed pairwise alignment analysis on the sequences’ inner-OTUs and neighbor-OTUs using BioEdit. Annotation of OTUs was performed by running BLASTn and MegaBlast searches of sequences using one representative sequence from each OTU against UNITE and the International Sequence Database (DDBJ/EMBJ/GenBank), separately. Sequences with ≥97% ITS identity to a known fungal species were identified as that species, and sequences with 90–97% identity to a known species were identified to the genus level^43^. When Blast results showed poor matches (<90% ITS identity), we treated the OTUs as unknown species. All sequences were submitted to the DNA Data Bank of Japan (DDBJ).

### Soil chemical analyses

Soil samples were air-dried for one week, and then stones and roots were removed. Half of each soil sample was then ground and passed through a 1-mm mesh screen for pH and electrical conductivity (EC) determination; the rest was passed through a 100- mesh sieve for soil elemental analyses. Soil pH and EC were measured using a FE20 pH meter (Mettler-Toledo Instrument Co., Ltd., Shanghai, China) and a DDS-307 Conductivity Meter (Shanghai REX Instrument Factory, Shanghai, China) after mixing the soil sample with deionized water at 1:2 and 1:5 ratios by volume, respectively. Soil organic matter (SOM) was determined by the Walkley–Black method^44^, by use of FeSO_4_ titration based on the oxidation of organic matter in soil by K_2_CrO_7_ and concentrated sulfuric acid. After digestion by sulfuric acid and perchloric acid, total phosphorus (TP) was determined by the molybdenum antimony colorimetric method^45^ using an ultraviolet spectrophotometer (UV-2450, Shimadzu Corporation, Kyoto, Japan) and total nitrogen (TN) was determined using the Kjeldahl method with a continuous flow analytical system (AutoAnalyzer3, Bran + luebbe, Hamburg, Germany).

### Data analysis

One-way analysis of variance (ANOVA) was used to compare the soil parameters (SOM, TN, TP, pH, EC) among the six sites, respectively. The ectomycorrhizal rate was defined as the percentage of ectomycorrhizal root tips in each root sample, and was also compared among the six sites by one-way ANOVA after log transformation. The relative abundance of a given EMF taxon in a root sample was calculated as its percentage in the ectomycorrhizal root tips. The relative abundance in each sample was pooled to calculate the relative abundance at a site. The frequency of each EMF taxon was the number of trees in which a given taxon was detected at each site.

To evaluate the potential species richness at each site and compare the species richness among the six sites, species accumulation curves were constructed using the rarefaction method and then extrapolated to the same sample size (*n* = 30) for those sites with <30 samples using EstimateS^46^. The richness estimators (Jackknife1, Jackknife2, and Chao2 estimators) and diversity indices (Shannon–Wiener *H’* and Simpson’s 1/*D*) were also calculated for each site using EstimateS. In addition, one-way ANOVA was performed on the richness and diversity indices among different communities. The Bray-Curtis dissimilarity index was also calculated for each site to measure their beta diversity using ‘vegdist’ in the vegan package of R program. The similarity in OTU composition between sites was calculated using the Morisita–Horn index based on the presence/absence data matrix. Cluster analysis of the six sites was performed based on the EMF OTU compositions using unweighted pair group method with arithmetic mean (UPGMA) method (Bray–Curtis distances, bootstrap = 999).

The mantel test was used to evaluate the effect of distance isolation through correlation between geographical distances and Bray–Curtis distances of EMF composition^47^. The geographical distances were obtained from GPS coordinates of the six sites using the function “distm” in the ‘geosphere’ package in R. The following statistical analyses were performed using the ‘vegan’ package in R. The effect of EMF composition on site ordination was illustrated by non-metric multidimensional scaling (NMDS, meta-MDS function) based on Bray–Curtis distances. The correlation of soil and climate parameters with NMDS ordination structure was tested by permutation tests (environmental fitting test, Envfit function, 999 permutations). Canonical correspondence analysis (CCA) was performed to determine the effects of precipitation, altitude, and soil parameters on EMF communities. Indicator species analysis was performed to evaluate the host and forest types preference of EMF-OTUs^48^,^49^. Point biserial correlation coefficient (*r*_pb_) was an available indices, which was computed between a quantitative variable of species abundance data and a binary variable indicating whether the site belongs to a site group combination under study, or not^27^. The detailed definition of *r*_pb_ index see De Cáceres and Legendre^48^. Two independent tests had been conducted for the preference of EMF OTUs to hosts and forest type. A higher *r*_pb_ value indicates a stronger association strength between EMF and hosts (forest types). Finally, the statistical significance of this relationship is tested using a permutation test. These statistical analyses were performed using the function multipatt () of the indicspecies package in R-language.

### Software

Data analysis was completed by EstimateS 9.1.0 and R version 3.2.0. Figures were plotted by Arcgis10.2 and R 3.2.0.

## Additional Information

**How to cite this article**: Long, D. *et al.* Ectomycorrhizal fungal communities associated with *Populus simonii* and *Pinus tabuliformis* in the hilly-gully region of the Loess Plateau, China. *Sci. Rep.* **6**, 24336; doi: 10.1038/srep24336 (2016).

## Supplementary Material

Supplementary Information

## Figures and Tables

**Table 1:** Ectomycorrhizal colonization and diversity indices for each sample site.

	QJ1 (Poplar) *n* = 15	QJ2 (Poplar) *n* = 15	YC1 (Poplar) n = 15	YC2 (Pine) *n* = 24	YA (Pine) *n* = 15	HL (Pine) n = 30
Ectomycorrhizal colonization	0.63 ± 0.15a	0.76 ± 0.15a	0.74 ± 0.09a	0.72 ± 0.16a	0.64 ± 0.08a	0.65 ± 0.11a
Observed richness/site	18	19	20	21	26	53
Observed richness/sample	3.27 ± 0.96a	3.07 ± 0.70a	3.07 ± 0.59a	2.79 ± 0.88a	3.60 ± 1.12a	4.90 ± 1.32b
Chao2 ± SD	19.04 ± 1.49a	20.33 ± 1.83a	26.72 ± 6.05a	23.4 ± 2.8 (23.32 ± 5.04a)	29.36 ± 3.17a	75.31 ± 11.86 (67.1 ± 17.17b)
Jack1	22.67 ± 1.76a	23.67 ± 1.76a	28.4 ± 2.29a	26.75 ± 3.46 (24.89 ± 3.11a)	34.4 ± 2.29a	77.17 ± 4.63 (58.21 ± 3.97b)
Jack2	20.56a	23.17a	32.89a	27.87 ± 2.61 (27.82 ± 5.6a)	34.96a	89.69 (71.66 ± 9.61b)
Shannon index H’	2.44a	2.74ab	2.54a	2.61 (2.47 ± 0.1a)	2.95ab	3.31 (3.08 ± 0.09b)
Simpson (1-1/D)	7.38a	13.1ab	9.49a	10.82 (9.61 ± 1.15a)	15.99b	13.3 (12.72 ± 1.82b)
Bray-Curtis dissimilarity index	0.74 ± 0.16	0.90 ± 0.16	0.85 ± 0.20	0.90 ± 0.18	0.93 ± 0.14	0.80 ± 0.15

The rarefied values of Chao2, Jack1, and Jack2 at the sample size of 15 are given for YC2 and HL.

Different letters refer to significant differences according to Tukey’s test at *P* < 0.05.

**Table 2:** Indicator species analysis showing host preference of EMF OTUs which had relative abundance >1% on any host species.

EMF-OTUs	*Populus simonii*^a^	*Pinus tabuliformis*^a^	*R*_pb_	*P* value	Signficance
*Geopora* sp.1	**0.105/0.378**	–	0.395	0.001	***
*Tomentella* sp.11	**0.136/0.267**	–	0.346	0.001	***
*Inocybe exilis*	**0.066/0.222**	–	0.303	0.001	***
*Inocybe* sp.3	**0.038/0.244**	–	0.291	0.001	***
*Inocybe pruinosa*1	0.046/0.133	–	0.234	0.004	**
*Tomentella* sp.20	0.061/0.155	–	0.231	0.002	**
*Inocybe* sp.2	0.027/0.111	–	0.224	0.010	**
*Tomentella fuscocinerea*1	0.045/0.111	–	0.214	0.008	**
*Tomentella* sp.6	0.047/0.111	–	0.200	0.012	*
*Tomentella* sp.2	0.035/0.111	–	0.186	0.007	**
*Cenococcum geophilum*2	–	**0.086/0.391**	0.438	0.001	***
*Suillus luteus*	–	**0.030/0.261**	0.305	0.001	***
*Amphinema* sp.1	–	0.046/0.188	0.291	0.005	**
*Tomentella* sp.21	–	0.029/0.145	0.234	0.006	**
*Geopora sp.*2	–	0.046/0.130	0.220	0.018	*
*Sebacina epigaea*2	–	0.024/0.116	0.208	0.021	*
*Tomentella* sp.10	–	0.050/0.116	0.183	0.032	*

^a^Values are relative abundance/frequency.

**Table 3:** Location, sampling, host, climatic, and soil characteristics of the study sites on the Loess Plateau.

Sites	QJ1	QJ2	YC1	YC2	YA	HL
Location	Qingjian County	Qingjian County	Yanchuan County	Yanchuan County	Yan’an City	Huanglong County
Latitude/ longitude	37°07′ N/110°04′ E	37°06′ N/110°17′ E	36°53′ N/110°06′ E	36°49′ N/110°14′ E	36°37′ N/109°27′ E	35°45′ N/109°54′ E
Vegetation zone	Forest-steppe	Forest-steppe	Forest-stepp	Forest-steppe	Forest-steppe transitional region	Forest
Vegetation type (tree ages)	Natural secondary poplar forest patch (40–50 years)	Natural secondary poplar forest patch (40–50 years)	Natural secondary poplar forest patch (40–50 years)	Artificial pine forest patch (40–45 years)	Artificial pine forest patch (40–45 years)	Natural secondary pure pine stand (30–40 years)
DBH (cm)^a^	11.5–35.6 (22.3)	10.2–37.2 (20.2)	11.3–34.7 (20.8)	10.4–18.5 (14.2)	11.0–33.1 (20.6)	16.8–35.5 (26.2)
Altitude (m)	940–1026	855–937	845–984	744–827	1014–1068	1481–1524
Precipitation (mm)	500	500	500	500	514	612
MAT (°C)	9.6	9.6	10.8	10.8	7.0	8.6
*Soil characteristics*^b^						
pH	8.04 ± 0.13b	8.47 ± 0.14d	8.24 ± 0.08c	8.37 ± 0.12cd	7.88 ± 0.20ab	7.76 ± 0.23a
EC (μS/cm)	209.51 ± 56.80b	133.91 ± 30.83a	131.34 ± 15.24a	109.41 ± 24.49a	146.85 ± 49.96a	191.23 ± 47.67b
SOM (g/kg)	12.73 ± 2.88a	13.71 ± 4.46a	15.85 ± 4.87a	15.17 ± 4.82a	10.23 ± 1.71a	66.69 ± 27.54b
TN (mg/kg)	714.68 ± 199.14a	735.01 ± 269.45a	887.53 ± 262.22a	760.50 ± 356.48a	435.65 ± 115.35a	3174.43 ± 1285.35b
TP (mg/kg)	523.13 ± 59.18c	499.77 ± 48.83bc	498.83 ± 29.96bc	463.69 ± 51.68b	404.04 ± 83.02a	467.96 ± 57.92b

MAT, mean annual temperature; EC, electronic conductivity; SOM, soil organic matter; TN, total nitrogen; TP, total phosphorus; DBH, diameter at breast height.

^a^Values are minimum-maximum with the mean in parentheses.

^b^The values are means ± standard errors. Values within each row followed by the same letter are not significantly different according to Tukey’s test at the 5% significance level.
