# Supplementary material for: Ectomycorrhizal fungal communities associated with Populus simonii and Pinus tabuliformis in the hilly-gully region of the Loess Plateau, China
Source: Sci Rep. 2016 Apr 11;6:24336. doi: 10.1038/srep24336 (PMC4827030; doi:10.1038/srep24336)
Supplement: Supplementary Information [file srep24336-s1.pdf]

Ectomycorrhizal fungal communities associated with *Populus simonii* and *Pinus tabuliformis* in the hilly-gully region of the Loess Plateau, China

Dongfeng Long[1], Jianjun Liu[2, 3*], Qisheng Han[1], Xiaobing Wang[1] & Jian Huang[1*]

[1] College of Forestry, Northwest A&F University, Yangling 712100, Shaanxi, China

[2] College of Landscape Architecture and Arts, Northwest A&F University, Yangling 712100, Shaanxi, China

[3] Ningxia Helan Mountain Forest Ecosystem Orientational Research Station, Yinchuan, 750000, Ningxia, China

* Correspondence and requests for materials should be addressed to J.H. (Jian Huang) (huangj@nwsuaf.edu.cn) or J.L. (Jianjun Liu) (ljj@nwsuaf.edu.cn)

Table S1 Identified EMF OTUs associated with Chinese pine (*Pinus tabuliformis*) and poplar (*Populus simonii*), and its relative abundance in each sampling site.

Code	OTUs	Accession number	Closest Blast match accession in GenBank/EMBJ/DDBJ/UNITE [a]	Identity [b]	Relative abundance(%)/relative frequency(%)					
					QJ1	QJ2	YC1	YC2	YA	HL
1	*Amanita phalloides*	LC013704	UDB000084 *Amanita phalloides*	641/654 (98%)						0.52/10.00
2	*Amphinema* sp.1	LC013706	UDB007425 *Amphinema*	507/521 (97%)				7.09/25.00		6.41/23.33
3	*Amphinema* sp.2	LC013708	KC152068.1 *Amphinema* sp.	490/521(94%)				1.78/16.67		
4	*Cenococcum geophilum*1	LC013709	JX145392.1 *Cenococcum geophilum*	1033/1055(98%)						0.08/10.00
5	*Cenococcum geophilum*2	LC013710	JN943885.1 *Cenococcum geophilum*	502/507(99%)					0.27/13.33	23.33/83.33
6	*Clavulina* sp.	LC013716	UDB000074 *Clavulina cinerea*	409/424 (96%)						1.20/6.67
7	*Cortinarius atrocoeruleus*	LC013717	UDB001011 *Cortinarius atrocoeruleus*	554/559 (99%)		2.98/13.33	0.71/6.67			
8	*Geopora arenicola*	LC013719	UDB011004 *Geopora arenicola*	582/585 (99%)			1.23/6.67			
9	*Geopora cervina*	LC013720	UDB018595 *Geopora cervina*	533/535 (99%)		3.39/20.00				
10	*Geopora* sp.1	LC013721	UDB011003 *Geopora sepulta*	547/609 (90%)	30.62/86.67	8.83/20.00	0.06/6.67			
11	*Geopora* sp.2	LC013727	UDB011006 *Geopora arenicola*	541/597 (91%)				18.51/37.51		
12	*Geopora* sp.3	LC013728	UDB017620 *Geopora arenicola*	560/599 (93%)					4.04/20.00	
13	*Geopora* sp.4	LC013729	UDB016155 *Geopora cervina*	528/574 (92%)		3.76/13.33				
14	*Geopora* sp.5	LC013731	FM206427.1 *Geopora* sp.	506/561(90%)	1.25/6.67					
15	*Hebeloma collariatum*	LC013732	UDB015489 *Hebeloma collariatum*	437/440 (99%)	0.85/6.67					
16	*Hebeloma leucosarx*	LC013733	UDB017591 *Hebeloma leucosarx*	654/661 (98%)		2.38/6.67				
17	*Hebeloma populinum*	LC013736	UDB000697 *Hebeloma populinum*	641/646 (99%)	8.61/20.00					
18	*Hebeloma* sp.	LC013737	UDB015489 *Hebeloma collariatum*	645/670 (96%)			1.21/6.67			
19	*Helvella lacunosa*	LC013738	KC122864.1 *Helvella lacunosa*	685/706(97%)						1.03/6.67
20	*Hydnobolites* sp.	LC013739	JX414186.1*Hydnobolites* sp. 11 RH-2012	511/544(94%)						1.00/10.00
21	*Inocybe decipiens*	LC013740	UDB017679 *Inocybe decipiens*	656/667 (98%)	2.51/13.33					
22	*Inocybe dulcamara*	LC013741	UDB001196 *Inocybe dulcamara*	512/519 (98%)			3.69/20.00			
23	*Inocybe dunensis*	LC013743	UDB017616 *Inocybe dunensis*	592/592 (100%)	2.63/6.67	4.10/13.33				
24	*Inocybe exilis*	LC013744	JX630516.1 *Inocybe exilis*	634/640(99%)	11.80/40.00	4.45/13.33	3.46/13.33			
25	*Inocybe fuscidula*	LC013747	JX129150.1 *Inocybe fuscidula var. fuscidula*	663/664(99%)					2.94/6.67	

26	*Inocybe ochroalba*	LC013748	HQ604369.1 *Inocybe ochroalba*	681/686(99%)	1.85/6.67					
27	*Inocybe pruinosa1*	LC013751	UDB017663 *Inocybe pruinosa*	610/617 (99%)		4.43/20.00	8.06/20.00			
28	*Inocybe pruinosa2*	LC013752	UDB015282 *Inocybe pruinosa*	566/578 (97%)		3.93/13.33				
29	*Inocybe pseudoreducta*	LC013753	EF644109.1 *Inocybe pseudoreducta*	644/655(98%)	4.37/13.33					
30	*Inocybe* sp.1	LC013757	UDB017682 *Inocybe decipiens*	415/459 (90%)			0.26/6.67	4.65/12.50		
31	*Inocybe* sp.2	LC013759	UDB015967 *Inocybe myriadophylla*	685/743 (92%)		3.81/13.33	3.26/20.00			
32	*Inocybe* sp.3	LC013764	UDB000101 *Inocybe godeyi*	639/700 (91%)	3.78/13.33	12.00/53.33	0.49/6.67			
33	*Inocybe* sp.4	LC013765	UDB000619 *Inocybe leptocystis*	380/416 (91%)				2.57/8.33		
34	*Inocybe* sp.5	LC013766	FJ904154.1 *Inocybe* cf. rimosa EL-2010d	583/624(93%)					3.19/13.33	
35	*Inocybe* sp.6	LC013767	HQ604622.1 *Inocybe rimosa var. rimosa*	638/687(93%)				3.14/4.17		
36	*Inocybe* sp.7	LC013768	EF644109.1 *Inocybe pseudoreducta*	643/684(94%)						1.58/6.67
37	*Lactarius deliciosus*	LC013769	UDB015792 *Lactarius deliciosus*	701/701 (100%)						1.72/10.00
38	*Pachyphlodes* sp.	LC013715	KJ720735.1 *Pachyphlodes sp.* 33 RH-2014	498/529(94%)						1.41/6.67
39	*Peziza* sp.1	LC013770	EU819535.1 *Peziza depressa*	583/608(96%)				0.07/4.17		
40	*Peziza* sp.2	LC013771	FN669234.1 *Peziza* sp. B276	568/577(98%)						1.06/6.67
41	*Peziza succosa*	LC013772	UDB015317 *Peziza succosa*	593/609 (97%)						0.07/3.33
42	*Pseudotomentella atrofusca*	LC013773	UDB016492 *Pseudotomentella atrofusca*	398/408 (97%)						0.23/3.33
43	*Pseudotomentella* sp.	LC013774	UDB011099 *Pseudotomentella tristis*	660/702 (94%)						0.36/3.33
44	*Rhizopogon roseolus*	LC013778	AM084707.1 *Rhizopogon roseolus*	665/687(97%)					0.93/6.67	
45	*Russula versicolor*	LC013779	UDB011297 *Russula versicolor*	647/652 (99%)						0.55/3.33
46	*Sebacina epigaea*1	LC013780	JQ665484.1 *Sebacina epigaea*	557/568(98%)						0.89/3.33
47	*Sebacina epigaea*2	LC013781	JQ665506.1 *Sebacina epigaea*	563/567(99%)					1.97/6.67	4.98/23.33
48	*Sebacina epigaea*3	LC013783	JQ665502.1 *Sebacina epigaea*	565/567(99%)						2.14/10.00
49	*Sebacina epigaea*4	LC013784	JQ665492.1 *Sebacina epigaea*	547/565(97%)						1.28/3.33
50	*Sebacina* sp.1	LC013786	UDB014142 *Sebacina*	570/589 (96%)					5.41/20.00	0.73/3.33
51	*Sebacina* sp.2	LC013787	UDB016419 *Sebacina*	524/571 (92%)					0.60/6.67	
52	*Sebacina* sp.3	LC013788	KF000410.1 *Sebacina epigaea*	542/588(92%)			3.64/6.67			
53	*Sebacina* sp.4	LC013789	UDB000118 *Sebacina incrustans*	561/583 (96%)					8.94/26.67	0.08/3.33
54	*Sebacina* sp.5	LC013792	UDB014118 *Sebacina incrustans*	562/580 (96%)						3.58/16.67

55	*Sebacina* sp.6	LC013793	UDB000774 *Sebacina*	556/596 (93%)					9.01/20.00	
56	*Sebacina* sp.7	LC013794	UDB016419 *Sebacina*	552/591 (93%)						2.33/6.67
57	*Sebacina* sp.8	LC013795	UDB016431 *Sebacina*	548/581 (94%)						1.24/3.33
58	*Sebacina* sp.9	LC013796	UDB016422 *Sebacina dimitica*	438/460 (95%)					6.51/20.00	
59	*Sebacina* sp.10	LC013797	JN129411.1 *Sebacina* sp. X3_15	551/599(92%)						1.00/3.33
60	*Sebacina* sp.11	LC013798	FN669246.1 *Sebacina* sp. B259	573/588(97%)						3.38/13.33
61	*Sebacina* sp.12	LC013800	UDB000975 *Sebacina epigaea*	493/527 (93%)					1.20/13.33	
62	*Sistotrema* sp.	LC013801	FN669255.1 *Sistotrema* sp. B216	542/586(92%)						1.52/6.67
63	*Suillus collinitus*	LC013802	UDB011905 *Suillus collinitus*	629/639 (98%)				2.61/20.83	0.10/6.67	
64	*Suillus luteus*	LC013805	UDB016610 *Suillus luteus*	659/661 (99%)				6.06/33.33		3.64/33.33
65	*Thelephora* sp.	LC013808	UDB000775 *Thelephora penicillata*	581/620 (94%)				2.95/4.17		
66	*Tomentella bresadolae*	LC013874	UDB016311 *Tomentella bresadolae*	621/637 (97%)						0.12/3.33
67	*Tomentella cinereoumbrina*	LC013811	UDB016491 *Tomentella cinereoumbrina*	619/634 (98%)			6.08//20.00		3.55/13.33	
68	*Tomentella ferruginea*	LC013812	UDB014253 *Tomentella ferruginea*	619/622 (99%)					9.63/20.00	1.32/3.33
69	*Tomentella fuscocinerea*1	LC013814	UDB016492 *Tomentella fuscocinerea*	619/630 (98%)	7.78/20.00		6.40/13.33			
70	*Tomentella fuscocinerea*2	LC013817	UDB016486 *Tomentella fuscocinerea*	593/605 (98%)		12.38/20.00		9.25/12.50		
71	*Tomentella lapida*1	LC013818	UDB016370 *Tomentella lapida*	625/638 (98%)						1.26/13.33
72	*Tomentella lapida*2	LC013819	JX145393.1 *Tomentella lapida*	624/628(99%)						1.09/3.33
73	*Tomentella lilacinogrisea*	LC013821	JX630533.1 *Tomentella lilacinogrisea*	624/628(99%)	4.36/13.33	3.19/6.67				
74	*Tomentella pilosa*	LC013850	UDB003324 *Tomentella pilosa*	579/592 (98%)	1.99/13.33					
75	*Tomentella* sp.1	LC013823	UDB018564 *Tomentella*	607/641 (95%)		4.63/20.00				1.81/10.00
76	*Tomentella* sp.2	LC013826	JX630675.1 *Tomentella badia*	608/640(95%)		2.34/6.67	7.91/26.67			
77	*Tomentella* sp.3	LC013827	UDB004953 *Tomentella*	570/600 (95%)				11.25/20.83	1.96/6.67	
78	*Tomentella* sp.4	LC013829	UDB018564 *Tomentella*	597/645 (93%)					2.56/13.33	1.21/3.33
79	*Tomentella* sp.5	LC013831	UDB018564 *Tomentella*	601/639 (94%)					1.90/13.33	
80	*Tomentella* sp.6	LC013833	UDB003309 *Tomentella cinerascens*	607/635 (96%)			10.00/33.33			
81	*Tomentella* sp.7	LC013834	HM189968.1 *Tomentella* sp.	580/644(90%)			1.03/6.67			
82	*Tomentella* sp.8	LC013835	UDB003311 *Tomentella*	603/640(94%)						0.76/3.33
83	*Tomentella* sp.9	LC013836	UDB003208 *Tomentella*	602/637 (95%)						1.66/6.67

84	*Tomentella* sp.10	LC013837	UDB000035 *Tomentella bryophila*	592/636 (93%)				5.60/12.50	9.82/33.33	
85	*Tomentella* sp.11	LC013843	UDB016492 *Tomentella fuscocinerea*	564/600 (94%)	3.72/6.67	10.09/20.00	23.92/53.33	1.14/8.33		
86	*Tomentella* sp.12	LC013846	UDB016492 *Tomentella fuscocinerea*	607/630 (96%)	2.41/13.33		8.08/13.33			2.39/10.00
87	*Tomentella* sp.13	LC013849	UDB016439 *Tomentella lateritia*	582/646 (90%)						0.42/3.33
88	*Tomentella* sp.14	LC013851	UDB011637 *Tomentella stuposa*	599/638 (94%)						0.12/3.33
89	*Tomentella* sp.15	LC013852	UDB016174 *Tomentella stuposa*	608/634 (96%)					2.19/6.67	1.26/6.67
90	*Tomentella* sp.16	LC013855	UDB001660 *Tomentella stuposa*	603/631 (96%)						3.71/20.00
91	*Tomentella* sp.17	LC013856	UDB018512 *Tomentella*	601/633 (95%)			6.29/13.33			
92	*Tomentella* sp.18	LC013858	UDB018564 *Tomentella*	594/639 (93%)	2.52/13.33					
93	*Tomentella* sp.19	LC013860	UDB018512 *Tomentella*	401/421 (95%)						2.03/6.67
94	*Tomentella* sp.20	LC013862	UDB018564 *Tomentella*	599/641 (93%)	3.70/20.00	11.03/20.00	4.24/6.67			
95	*Tomentella* sp.21	LC013866	UDB003328 *Tomentella*	597/624 (96%)					3.61/13.33	4.89/26.67
96	*Tomentella* sp.22	LC013869	UDB014248 *Tomentella muricata*	570/599 (95%)		1.21/6.67		12.11/20.83		0.43/3.33
97	*Tomentella* sp.23	LC013871	UDB014248 *Tomentella muricata*	567/601 (94%)				0.51/4.17		
98	*Tomentella* sp.24	LC013872	UDB018515 *Tomentella*	595/603 (99%)					2.69/13.33	
99	*Tomentella* sp.25	LC013873	UDB020325 *Tomentella*	600/635 (94%)						0.38/3.33
100	*Tomentella* sp.26	LC013809	UDB014253 *Tomentella ferruginea*	608/635 (96%)						0.92/3.33
101	*Tricholoma argyraceum*	LC013875	UDB000782 *Tricholoma argyraceum*	656/661 (99%)				0.12/4.17		
102	*Tricholoma terreum*	LC013876	AF377212.1 *Tricholoma terreum*	666/673(99%)					8.47/20.00	1.03/3.33
103	*Tuber* sp.1	LC013878	JF748075.1 Uncultured *Tuber*	428/448(96%)					0.67/6.67	
104	*Tuber* sp.2	LC013879	UDB015182 *Tuber rufum*	407/452 (90%)						1.71/6.67
105	*Tuber* sp.3	LC013881	UDB016154 *Tuber rufum*	397/439 (90%)	5.24/13.33			0.60/8.33		
106	Unknown 1	LC013883	JF908102.1 *Inocybe decemgibbosa*	381/443(86%)				5.67/8.33		
107	Unknown 2	LC013884	EU563476.1 Uncultured Pyronemataceae	518/596(87%)						1.06/3.33
108	Unknown 3	LC013885	AF289074.1 *Pulvinula constellatio*	478/561(85%)		1.07/6.67				
109	Unknown 4	LC013886	FM206478.1 *Tarzetta catinus*	408/469(87%)				3.81/8.33		
110	Unknown 5	LC013887	DQ974820.1 *Tarzetta* sp. src844	466/589(79%)						0.77/3.33
111	Unknown 6	LC013888	UDB015296 *Inocybe cincinnata*	587/662 (88%)						0.35/3.33
112	Unknown 7	LC013889	EU819461.1 *Peziza ostracoderma*	544/611(89%)				0.53/4.17		
113	Unknown 8	LC013890	DQ974736.1 *Hysterangium* sp. src642	381/442(86%)						0.46/3.33

114	Unknown 9	LC013775	GQ281482.1 Pyronemataceae sp. JW44a	512/556(92%)	2.02/6.67	1.50/10.00
115	Unknown 10	LC013777	GQ281480.1 Pyronemataceae sp. JW76a	564/592(95%)	5.84/13.33	

[a] Closest matched Blast results with informative species and genera were used

[b] Aligned/query portion length (bp) (similarity, %)

Table S2 The number of shared EMF OTUs (lower triangle matrix) and the Morisita–Horn similarity index (upper triangle matrix) between different sites.

	QJ1	QJ2	YC1	YC2	YA	HL
QJ1		0.378	0.368	0.103	0	0.028
QJ2	7		0.462	0.15	0	0.056
YC1	7	9		0.098	0.043	0.027
YC2	2	3	2		0.128	0.081
YA	0	0	1	3		0.253
HL	1	2	1	3	10	

Table S3. Effects of precipitation, altitude, and soil parameters on ectomycorrhizal fungal (EMF) species distribution in the whole EMF community based on canonical correspondence analysis (CCA).

Factors	R^2	P-value	CCA1	CCA2
PRE	0.698	***	−0.974	0.227
ALT	0.620	***	−0.982	0.190
SOM	0.509	***	−0.921	0.390
TN	0.484	***	−0.912	0.410
pH	0.412	***	0.999	−0.024
TP	0.379	***	0.640	0.769
EC	0.048	0.069	−0.851	0.525

Significance: *** $P < 0.001$. P values based on 999 permutations.

PRE, precipitation; ALT, altitude; SOM, soil organic matter; TN, total nitrogen; TP, total phosphorus; EC, electronic conductivity.

Table S4. Effects of soil nutrients, climatic factors and host on EMF community structure at six sites based on non-metric multidimensional scaling (NMDS) ordination with the environmental fitting test.

	NMDS1	NMDS2	R^2	*P* values	Significance
Host	0.947	-0.320	0.771	0.001	***
ALT	0.456	0.890	0.452	0.001	***
PRE	0.564	0.825	0.444	0.001	***
pH	-0.582	-0.813	0.365	0.001	***
SOM	0.548	0.836	0.267	0.001	***
TN	0.514	0.858	0.249	0.001	***
TP	-0.934	0.356	0.168	0.001	***
DBH	0.078	0.997	0.121	0.001	***
EC	0.103	0.995	0.042	0.084	

Significance: ***, $P < 0.001$. *P* values based on 999 permutations.

ALT, altitude; PRE, precipitation ;TN, total nitrogen; TP, total phosphorus; SOM, soil organic matter; EC, electronic conductivity.

Table S5 Indicator species analysis showing significant preference of EMF-OTUs to forest types

EMF-OTUs	Forest-steppe zone[a]	Transitional zone[a]	Forest zone[a]	R_{pb}	*P* values
Geopora sp.1	**0.078/0.246**			0.353	0.006
Tomentella sp.11	**0.010/0.203**			0.329	0.006
Sebacina sp.9		**0.075/0.200**		0.374	0.003
Sebacina sp.4		**0.085/0.267**	0.001/0.003	0.370	0.001
Tomentella ferruginea		**0.094/0.200**	0.008/0.003	0.329	0.005
Tomentella sp.10	0.017/0.043	**0.097/0.333**		0.324	0.004
Sebacina sp.1		**0.060/0.200**	0.007/0.003	0.313	0.004
Sebacina sp.6		**0.110/0.200**		0.312	0.003
Tricholoma terreum		**0.088/0.200**	0.014/0.003	0.311	0.004
Geopora sp.3		**0.035/0.200**		0.264	0.003
*Cenococcum geophilum*2		0.003/0.133	**0.222/0.833**	0.796	0.001
Tomentella sp.16		0.028/0.003	**0.013/0.003**	0.352	0.004
Sebacina sp.5			**0.027/0.167**	0.324	0.010

[a] Values are relative abundance/frequency.
